# 3D hUC-MSC spheroids exhibit superior resistance to autophagy and apoptosis of granulosa cells in POF rat model

**DOI:** 10.1530/REP-23-0496

**Published:** 2024-07-13

**Authors:** Wenjie Dai, Hong Yang, Bo Xu, Tiantian He, Ling Liu, Zhen Zhang, Liyang Ding, Xiuying Pei, Xufeng Fu

**Affiliations:** 1 Key Laboratory of Fertility Preservation and Maintenance of Ministry of Education, School of Basic Medical Sciences, Ningxia Medical University, Yinchuan, China

## Abstract

**In brief:**

This study reveals that orthotopic transplantation of 3D hUC-MSC spheroids is more effective than monolayer-cultured hUC-MSCs in improving POF and distinctly reducing oxidative stress through the paracrine effect, thereby preventing apoptosis and autophagy of GCs.

**Abstract:**

Premature ovarian failure (POF) is a common reproductive disease in women younger than 40 years old, and studies have demonstrated that the application of human umbilical cord mesenchymal stem cells (hUC-MSCs) is a promising therapy strategy for POF. Given the previously established therapeutic advantages of 3D MSC spheroids, and to evaluate their effectiveness, both 3D hUC-MSC spheroids and monolayer-cultured hUC-MSCs were employed to treat a cyclophosphamide-induced POF rat model through orthotopic transplantation. The effects of these two forms on POF were subsequently assessed by examining apoptosis, autophagy, and oxidative damage in ovarian granulosa cells (GCs). The results indicated that hUC-MSC spheroids exhibited superior treatment effects on resisting autophagy, apoptosis, and oxidative damage in GCs compared to monolayer-cultured hUC-MSCs. To further elucidate the impact of hUC-MSC spheroids *in vitro*, a H_2_O_2_-induced KGN cells model was established and co-cultured with both forms of hUC-MSCs. As expected, the hUC-MSC spheroids also exhibited superior effects in resisting apoptosis and autophagy caused by oxidative damage. Therefore, this study demonstrates that 3D hUC-MSC spheroids have potential advantages in POF therapy; however, the detailed mechanisms need to be further investigated. Furthermore, this study will provide a reference for the clinical treatment strategy of POF.

## Introduction

Premature ovarian failure (POF) is a disease of the female reproductive system that affects approximately 2% of women under 40, resulting in amenorrhea, low estrogen levels, and follicle failure (Chon *et al.* 2021). Infertility is the most direct consequence of POF, while other complications such as cardiovascular disease, osteoporosis, and sexual dysfunction are also associated with POF (Liu *et al.* 2021, Wang *et al.* 2022*a*,*b*). Therefore, POF has a significant effect on the reproductive health and quality of life of women. Current research suggests that the primary cause of POF is low ovarian reserve function due to atresia of the dominant follicles and failure of the primordial follicles (PrFs) in the ovary due to oxidative stress (Sun *et al.* 2018, Jiang *et al.* 2019*a*, *b*, Asadi *et al.* 2022). Granulosa cells (GCs) play a crucial role in follicular development and maturation by producing estradiol and other maturation-promoting factors (Zhu *et al.* 2022). Studies have revealed that apoptosis and excessive autophagy of GCs caused by oxidative stress are the primary causes of follicular dysfunction and POF occurrence (Shen *et al.* 2018, Xu *et al.* 2022). Autophagy is a physiological process that helps in maintaining cellular homeostasis; however, persistent oxidative stress can lead to excessive autophagy, resulting in cell death (Zhou *et al.* 2019). Normal autophagy is essential for the selection of dominant follicles and follicle development in GCs, whereas excessive autophagy can cause follicle atresia (Liu *et al.* 2023, Yin *et al.* 2020). To maintain the homeostasis of GCs, the antioxidant system composed of glutathione (GSH), superoxide dismutase (SOD), and catalase (CAT) plays a vital role in resisting oxidative stress. Therefore, to improve ovarian function in POF, it is necessary to explore efficient methods to prevent GC apoptosis and excessive autophagy induced by oxidative stress.

Due to the limitations of current treatments, stem cell therapy has emerged as a promising strategy for treating POF (Wang *et al.* 2022*a*,*b*). The advantages of mesenchymal stem cells (MSCs) include multi-directional differentiation, low immunogenicity, and immunomodulation (Lin *et al.* 2022). Additionally, MSCs secrete a variety of cytokines, such as VEGF, EGF, HGF, IGF, TNF-α, NGF, and TrkA, which regulate the immune system and repair damaged tissues (Li *et al.* 2021*a*,*b*). Multiple lines of clinical transplantation trials have confirmed the effectiveness of human umbilical cord-derived mesenchymal stem cells (hUC-MSCs) in the treatment of a variety of diseases, demonstrating their promising clinical applications (Hernandez *et al.* 2021, Litvinova *et al.* 2022). hUC-MSCs are superior in the treatment of several diseases, including POF (Umer *et al.* 2023). Additionally, hUC-MSCs have high proliferation ability, powerful secretion ability, low immunogenicity, and few ethical concerns due to their source from discarded perinatal tissue (Huang *et al.* 2016, Hai *et al.* 2022, Umer *et al.* 2023). Therefore, the application of hUC-MSCs is considered one of the optimal strategies for POF treatment. However, studies have shown that MSCs transplanted intravenously have a very low success rate for ovarian tissue (Belmadi *et al.* 2015, Kim *et al.* 2020). Orthotopic transplantation can increase the number of hUC-MSCs in ovarian tissue treatment, thereby improving the therapeutic effect (Huang *et al.* 2022). Numerous studies have demonstrated that 3D MSC spheroids can maintain their survival rate and paracrine capacity in injured tissue while also more accurately simulating the real situation in tissue (Xu *et al.* 2016, Jiang *et al.* 2019*a*, *b*, Yuan *et al.* 2022). Therefore, we hypothesized that orthotopic transplantation of 3D hUC-MSC spheroids could increase the therapeutic effect on POF.

In this study, the therapeutic effects of monolayer-cultured hUC-MSCs and 3D hUC-MSC spheroids on the cyclophosphamide (CTX)-induced POF rat model were compared by evaluating the apoptosis and autophagy of GCs. Studies, including ours, have shown that CTX-induced POF is due to oxidative damage of GCs (Chen *et al.* 2022, Dai *et al.* 2023). Subsequently, the levels of autophagy and apoptosis, and the antioxidant effects of the two forms of hUC-MSCs on H_2_O_2_-induced KGN cells were compared *in vitro* to clarify the therapeutic effects of hUC-MSCs on POF (Shen *et al.* 2017, 2018). This study will provide a novel strategy and a reference for the clinical application of hUC-MSCs in the treatment of POF.

## Materials and methods

### Isolation and identification of hUC-MSCs

The umbilical cords of full-term neonates were collected after receiving ethical approval from the Ethical Review Committee of Ningxia Medical University and after the cord donors and their families signed the informed consent forms. The cord tissue was sterilized with 75% alcohol for 30 s, placed in a PBS solution, and transferred within 1 h to a clean bench. The redundant fatty tissue and blood vessels were removed from the tissue and dissected into 0.5 cm × 0.5 cm pieces before being cultured in a sterile 10 cm plastic petri dish with 10 mL of low glucose DMEM (Gibco) containing 10% fetal bovine serum (FBS, Gibco) and 1% penicillin–streptomycin (Gibco) in an incubator at 37°C and 5% CO_2_. The medium was replaced every 48 h. A large number of fibroblast-like cells appeared around the tissue clump 1 week later. After the removal of tissue fragments, the obtained primary cells were passaged with 0.25% trypsin (Gibco), and the primary hUC-MSCs were passaged in new dishes at a dilution of 1:3. The morphology, surface markers, and differentiation potentials of the hUC-MSCs were determined in the third passage (Fu *et al.* 2020).

### Determination of differentiation potential of hUC-MSCs

The cells were seeded at 3 × 10^4^ cells/cm^2^ density in six-well plates, and 2 mL complete medium were added to each well. The plates were incubated at 37°C and 5% CO_2_. After 48 h, the three-lineage differentiation induction medium was changed, and fresh induction medium was added every 2 days. After 21 days of continuous cultivation, the induced cells were fixed with 4% paraformaldehyde and washed twice with PBS following differentiation. Oil red O, Alizarin red S, and Alcian blue were then used to stain the differentiation of adipogenesis, osteogenesis, and chondrogenesis, respectively. The staining solution was discarded, and the cells were washed three times with PBS, then observed under a microscope and photographed. The details have been described in our previous study (Fu *et al.* 2020).

### Surface markers identification of hUC-MSCs

The expression of cell surface markers was evaluated using a Human MSCs Analysis Kit (BD Biosciences, USA) on a C6 flow cytometer (BD Biosciences) at the third passage. Briefly, 2 × 10^6^ hUC-MSCs were collected and resuspended with 500 μL of PBS (containing 3% FBS, PBSF). Then, 5 µL (10 µg/µL) of human monoclonal antibodies against positive markers (CD44, CD73, CD90, and CD105) and hematopoietic markers (including CD34, CD45, CD14, CD19, and HLA-DR) were incubated for 30 min at room temperature, according to the manufacturer’s instructions. Unbound antibodies were washed with PBS, and the cells were resuspended in 500 μL of PBSF. The labeled MSCs were then assayed by FACS Aria II (BD Biosciences), and 10^4^ events were recorded. The data were analyzed using the built-in software of the instrument. The details have been described in our previous study (Fu *et al.* 2020).

### Animal experiments

A total of 20 inbred female specific pathogen-free Sprague-Dawley (SD) rats, 5 weeks (mean body weight 180 ± 20 g), were obtained from the Experimental Animal Center of Ningxia Medical University. All handling and animal care procedures were performed according to the guidelines of the National Institutes of Health of China and were approved by the Medical Ethics Committee of Ningxia Medical University (2019-190). All animals were given access to food and sterile water *ad libitum* under a controlled temperature of 22 ± 2°C and a 12 h light–12 h darkness cycle. The rats were randomly divided into the Control (Control, *n* = 5) and POF (POF, *n* = 15) groups. The estrus cycle was detected by vaginal exfoliated cell smear, and the estrus cycle of 96–120 h was selected for the subsequent establishment of animal models. The rats in the POF group were intraperitoneally injected with CTX (50 mg/kg/day for the first day, followed by 8 mg/kg/day for 14 days), while the rats in the Control were injected with an equal amount of saline (Dai *et al.*, 2023). After successfully establishing the POF group, rats were randomly divided into three groups (*n* = 5 in each group): the POF+Saline group (for control of treatment groups), the POF+MSCs (M) group (for transplanting monolayer-cultured hUC-MSC suspension), and the POF+MSCs (S) group (for transplanting 3D-cultured hUC-MSC spheroids), while the normal control group continued to be fed. After the fifth passage, hUC-MSCs were collected and washed three times with saline. At 48 days after the orthotopic transplantation of MSCs, rats were euthanized, and ovaries were collected for ovarian parameters and function evaluations. Based on nine consecutive days of estrus cycle measurements, blood was collected from the tail vein during anoestrus for hormone measurements.

### Preparation of 3D hUC-MSC spheroids and orthotopic transplantation

The obtained third-generation hUC-MSCs were resuspended in fresh medium, and the concentration was adjusted to 1 × 10^6^ cells/mL. About 25 μL of the cell suspension was evenly dripped onto the lid of a 10 cm sterile petri dish, which was then gently turned over and inverted onto a petri dish with PBS. The setup was gently placed into the incubator for 48 h. The prepared 3D hUC-MSC spheroids were collected for subsequent co-culture experiments (Supplementary Fig. 1A, see section on supplementary materials given at the end of this article). After routine disinfection, the skin and muscle were cut from the dorsal costovertebral angle, and the ovaries were exposed in a sterile environment. The ovaries and fallopian tubes were removed, and the monolayer-cultured hUC-MSC suspension or 3D-cultured hUC-MSC spheroids were injected steadily into the ovary. The ovary was sutured with an ophthalmic needle (1/2 arc, 8 × 20, Shanghai Medical Suture Needle Co. Ltd), and the ovary and fallopian tube were returned slowly to the abdominal cavity, followed by the suturing of the muscles and skin. The rats in the POF+MSCs (M) group were injected with monolayer-cultured hUC-MSC suspension (1 × 10^6^ cells/20 μL) by orthotopic transplantation into the ovaries. The rats in the POF + MSCs (S) group were injected with 3D-cultured hUC-MSC spheroids (equal with 10^6^ cells), whereas the Control and POF + Saline groups were injected with 20 μL of saline. Subsequent assays were detected after 48 days of hUC-MSC transplantation (Supplementary Fig. 1B).

### Histology evaluation and follicle counting

The ovaries of rats in each group were first removed and fixed with 4% paraformaldehyde in a refrigerator at 4°C for 24 h, followed by gradient alcohol dehydration and embedding with paraffin. Secondly, the ovaries were serially sectioned at a thickness of 5 μm, and multiple sections were selected at five intervals for hematoxylin and eosin staining. Finally, ovarian morphology was observed and photographed using microphotographic equipment (Tissue Nostic, Austria). The total number of PrFs, primary follicles (PFs), secondary follicles (SFs), mature follicles, and atretic follicles (ATFs) were counted, avoiding double counting by different markers. The classification of follicular stages was described in our previous study (Fu *et al.* 2020). PrFs were defined as an oocyte surrounded by a layer of squamous (flattened) GCs. PFs possessed an oocyte surrounded by a single layer of cuboidal GCs. SFs were surrounded by more than one layer of cuboidal GCs, with no visible antrum. Mature follicles, also named antral follicles (ANFs), have a large follicular cavity and a thin stratum granulosum, and GCs no longer proliferate. ATFs lost and shed the GCs into the follicular cavity, and the oocyte had nuclear contraction (Dai *et al.* 2024).

### Immunohistochemistry

The paraffin sections of the ovary were deparaffinized with xylene and rehydrated with gradient ethanol. After heating and repairing with sodium citrate solution, all sections were blocked with 3% H_2_O_2_ and 10% goat serum according to the kit’s instructions. The sections were incubated with the primary antibody overnight at 4°C, washed with PBS, subsequently incubated with a horseradish peroxidase-labeled secondary antibody for 2 h, and then washed with PBS. After staining with diaminobenzidine, the color development reaction was terminated by adding water, the nuclei were stained with hematoxylin for 2 min, and the sections were sealed with neutral resin. After drying, the positive cells were photographed with photomicrograph equipment (Tissue Gnostics, Austria). CYP19A1 (A12684) was purchased from ABclonal Technology Co., Ltd, China. 4-Hydroxynonenal (4-HNE) was purchased from Bioss Biotech Co. Ltd, China. All primary antibodies were diluted at 1:200.

### Determination of serum hormones

Follicle-stimulating hormone (FSH), estradiol (E2), and luteinizing hormone (LH) levels were determined according to the Elabscience ELISA kit instructions. The serum in each group of rats was diluted 1:10. In total, 96-well plates containing antibodies from the kit were prepared, and the diluted serum was added gently and accurately, then co-incubated in a thermostat at 37°C for 2 h. After completion of the reaction, a termination solution was added, and serum hormone levels were determined using an enzyme labeling instrument (Thermo Fisher Scientific). Three serum samples in each group were tested.

### Cell culture and treatment

The human ovarian granulosa cell line KGN was purchased from the Shanghai Cell Bank of the Chinese Academy of Sciences. KGN cells were cultured in DMEM/F12 medium supplemented with 10% FBS and 1% penicillin/streptomycin, and incubated at 37°C and 5% CO_2_. The KGN cells were planted in a six-well plate at a density of 2.5 × 10^5^ cells/well and cultured for 24 h. Three or six hUC-MSC spheroids, or an equal number of monolayer cultured hUC-MSCs, were co-cultured with the KGN cells for 48 h in a transwell system. Four hours before KGN cell collection, H_2_O_2_ was supplemented at a working concentration of 100 μM. Finally, the KGN cells were used for subsequent determination (Shen *et al.* 2017, 2018).

### Isolation, culture, and characterization of GCs

Following treatment with hUC-MSCs, female 3-week-old SD rats were anesthetized and euthanized to harvest their ovaries. Preovulatory follicles within the ovaries were subsequently punctured under a stereomicroscope to isolate GCs. The collected cells were then washed and cultured in a complete DMEM/F12 medium (VivaCell, Shanghai, China) supplemented with 10% FBS (Gibco) at 37°C and 5% CO_2_. Upon subculture, purification, and characterization, the primary GCs were utilized for subsequent experimentation (Supplementary Fig. 2A and B).

### Determination of oxidative stress level

The malondialdehyde (MDA, A003-4-1) and glutathione (GSH, A006-2-1) in ovarian tissue and KGN cells were measured using commercial kits according to the manufacturer’s instructions (Nanjing Jiancheng Institute of Bioengineering, China). Briefly, tissue and cell samples were homogenized with an ultrasonic crusher and then centrifuged at 13,000 ***g*** for 15 min at 4°C to obtain the supernatant. Subsequently, the corresponding reagents were added and mixed with the supernatant according to the manufacturer’s instructions. In terms of the detection principle, the reaction of MDA with thiobarbituric acid showed a red color, and the absorbance at 532 nm was detected; the reaction of GSH with dithio-dinitrobenzoic acid was yellow, and the absorbance at 405 nm was detected. The protein concentration was detected by the BCA protein assay kit. Our previous study has described the detailed procedures (Xu *et al.* 2023).

### Measurement of cell viability

The cell viability assay kit (CCK-8) (Beyond Biotech, China) was used according to the manufacturer’s instructions. Briefly, KGN cells were seeded at a density of 8000 cells/well in a 96-well plate for 24 h, serial concentrations of H_2_O_2_ were added, and the cells were cultured at 37°C, 5% CO_2_ for 4 h before being incubated with 10% (v/v) kit reagent for 2 h. The absorbance of each well was measured at 450 nm using a microplate reader (Thermo Fisher Scientific). The mean of three independent tests was calculated to determine the effect of H_2_O_2_ on viability.

### Western blotting

Ovary tissues or KGN cells were treated on ice for 30 min with radioimmunoprecipitation assay lysis buffer containing a protease inhibitor. After homogenization, the protein extraction solution was harvested by centrifuging at 12,000 ***g*** for 15 min at 4°C. The total protein concentration of each sample was determined using the BCA protein assay kit (KeyGEN Biotech Co., Ltd, Jiangsu, China). After denaturation at 100 °C for 10 min, 40 μg of each sample was separated by SDS-PAGE at 100 V. The separated proteins were then transferred to PVDF membranes by immunoblotting. The PVDF membrane was blocked for 30 min with 0.05 g/mL defatted milk in PBS containing 1% (v/v) Tween 20 (PBST). After washing with PBST, the primary antibody was incubated overnight at 4°C, followed by a 2 h incubation with the secondary antibody. Finally, the blotting was detected using an ECL kit (KeyGEN Biotech Co., Ltd) on a fluorescence detection instrument (Bio-Rad). All primary antibodies were diluted at 1:1000, whose sources were provided in the supplementary material (see section on supplementary materials given at the end of this article). Representative blotting images were selected from at least three independent replicate experiments.

### KI67 immunofluorescence

The KGN cells (1.5 × 10^4^) were seeded into a well of a 24-well plate pre-positioned with cell crawlers (round coverslip), and then co-cultured with hUC-MSC spheroids and treated with H_2_O_2_. After co-culturing, the medium was removed, and 4% paraformaldehyde was applied for 10 min. Subsequently, the cells were washed three times with PBS containing 0.5% Triton and blocked with 10% goat serum for 30 min. The KI67 primary antibody, diluted 1:200 was added and incubated at 37°C for 2 h. The cells were then washed with PBS and treated with 1% BSA diluted fluorescent secondary antibody, incubated at 37°C for 1 h, and stained with DAPI for 10 min. After rinsing with PBS and sealing with an anti-fluorescence quenching reagent, the images were captured using a confocal microscope (Nikon).

### Statistical analysis

ImageJ software (National Institutes of Health) was used to quantify the target protein and the internal reference protein to quantify the protein expression. All data were represented as mean ± s.d. using GraphPad Prism 8.0 software. Data were tested for normality prior to ANOVA or *t*-tests using GraphPad Prism 8.0 software. Various factors were compared using two-way ANOVA, and multiple samples were compared using one-way ANOVA with the minimum significant difference (LSD) method. A *P* value less than 0.05 was considered statistically significant.

## Results

### Isolation, culture, and identification of hUC-MSCs

**Figure 1:**
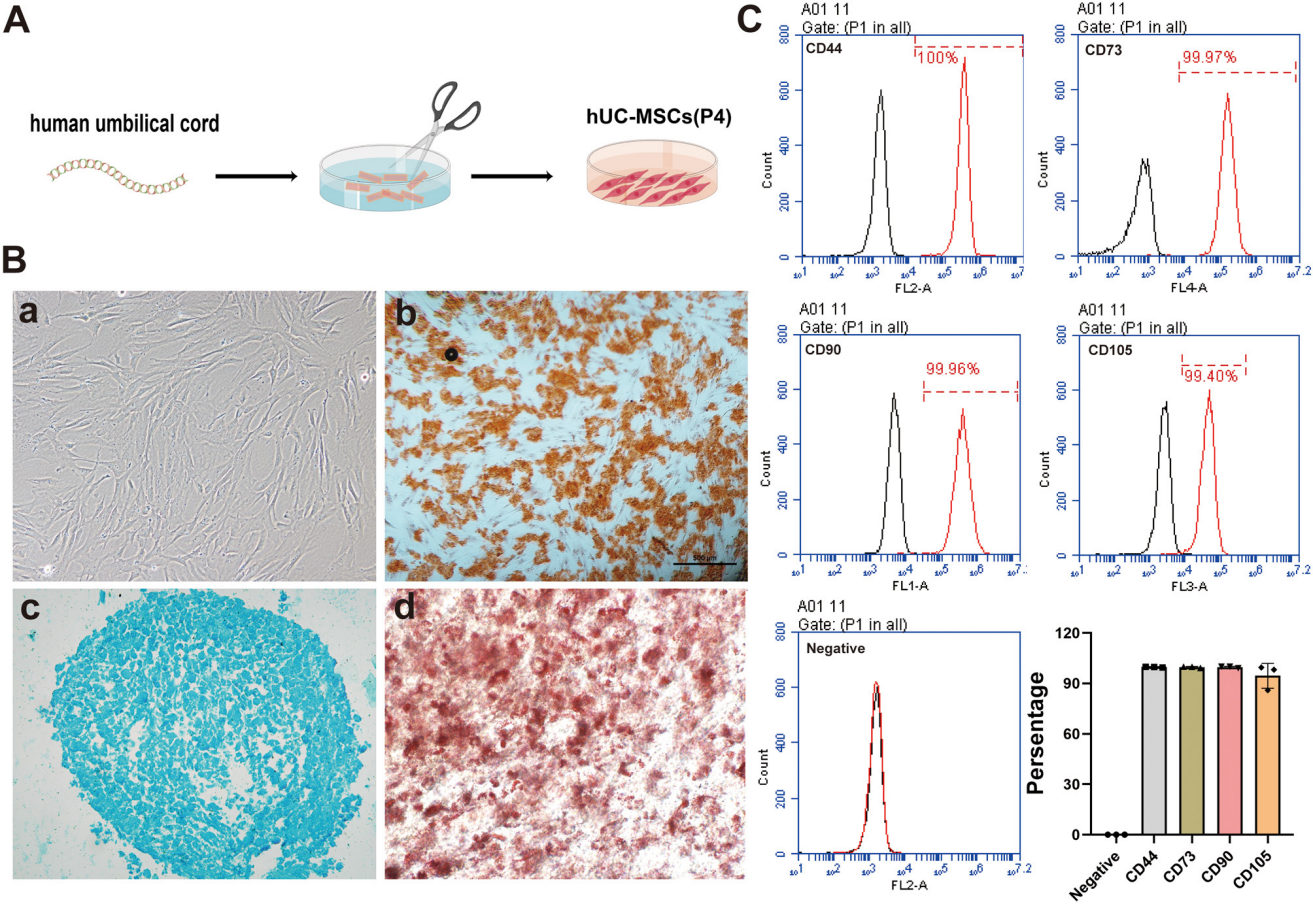
Isolation, culture, and identification of hUC-MSCs. (A) Schematic diagram illustrating the processing of umbilical cord tissue. (B) Morphology (a), osteogenesis (b), chondrogenesis (c), and adipogenesis (d) of hUC-MSCs. (C) Detection of positive markers (CD44, CD90, CD105, and CD73) and hematopoietic markers in hUC-MSCs using flow cytometry, with results presented in a quantitative statistical histogram.

The umbilical cord tissue was sectioned and cultured in an incubator (Fig. 1A). After 1 week, spindle-shaped, polygonal, or stellate cells appeared at the periphery of tissue pieces. The cells were passaged to the third generation, and their morphology was identified; the results showed long spindle-shaped and monolayer vortex-shaped cells (Fig. 1B, a). After adipogenic, osteogenic, and chondrogenic inducing differentiation for 21 days, hUC-MSCs have the potential for osteogenesis, chondrogenesis, and adipogenesis differentiation, which were identified by specific dyes (Fig. 1B, b–d), respectively. The surface marker expression of hUC-MSCs at passage three was identified by flow cytometry. The results showed that more than 98% of hUC-MSCs expressed CD44, CD90, CD73, and CD105, but none expressed the hematopoietic markers of the kit (Fig. 1C). These results suggested that the isolated cells conformed to the standards of the International Society for Cell Therapy and could be used for subsequent studies.

### hUC-MSCs improved the pathological characteristics of the POF rat model

**Figure 2:**
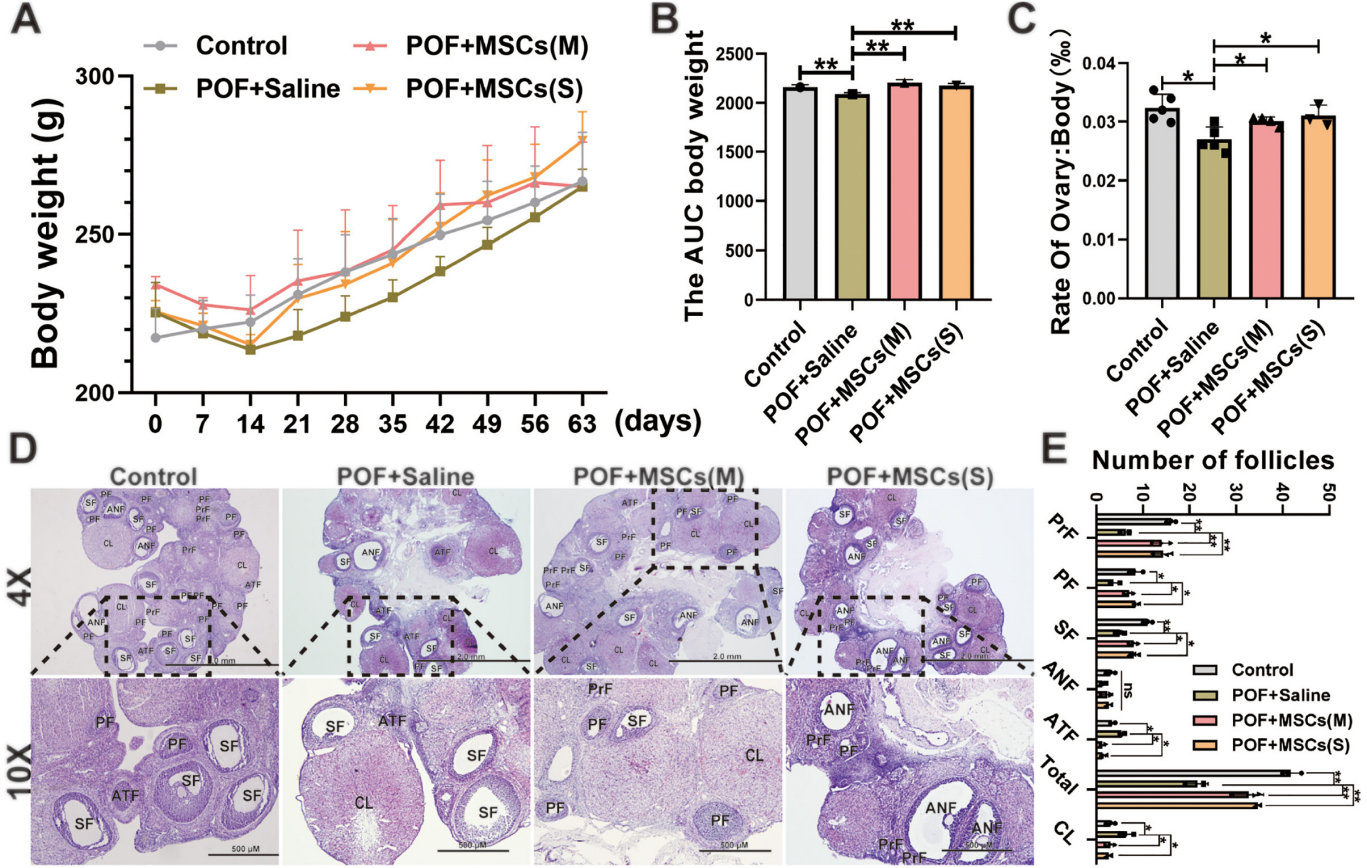
Effect of orthotopic transplantation of hUC-MSCs on POF rat model. (A) Changes in the body weight of rats during modeling and treatment were observed. (B) The area under the curve of body weight was calculated. (C) The ovarian organ coefficient of rats in each group was determined. (D) Ovarian histology was examined after the transplantation of monolayer hUC-MSCs and hUC-MSC spheroids. (E) The number of follicles at different stages was recorded. *P < 0.05, **P < 0.01; ANF, antral follicles; ATF, atretic follicle; ; CL, corpus luteum; SF, secondary follicle;PF, primary follicle; PrF, primordial follicle. Statistical tests for B and C involved one-way ANOVA followed by Dunnett’s test; for D and E, two-way ANOVA followed by Dunnett’s test.

The weight gain of rats in the POF group was significantly slower than that of the Control group during the modeling period, whereas the weight gain of the hUC-MSCs orthotopic transplantation groups was significantly greater than that of the POF+Saline group (Fig. 2A and B). Subsequently, the ovarian organ coefficient results showed that the POF+Saline group had a significantly lower coefficient than the Control group, whereas the POF+MSCs (M) and POF+MSCs (S) groups were restored considerably in comparison to the POF+Saline group (Fig. 2C). Compared to the Control group, ovarian histology revealed that the number of PrF, PFs, and SFss was substantially reduced in the POF+Saline group, while the number of ATFs was increased. After hUC-MSCs transplantation, the number of follicles at different stages was restored in the POF+MSCs (M), and POF + MSCs (S) groups compared to the POF+Saline and Control groups (Fig. 2D and E). These results indicated that two forms of hUC-MSCs transplantation could significantly improve the POF rat model.

### hUC-MSCs improved ovarian function in the POF rat model

**Figure 3:**
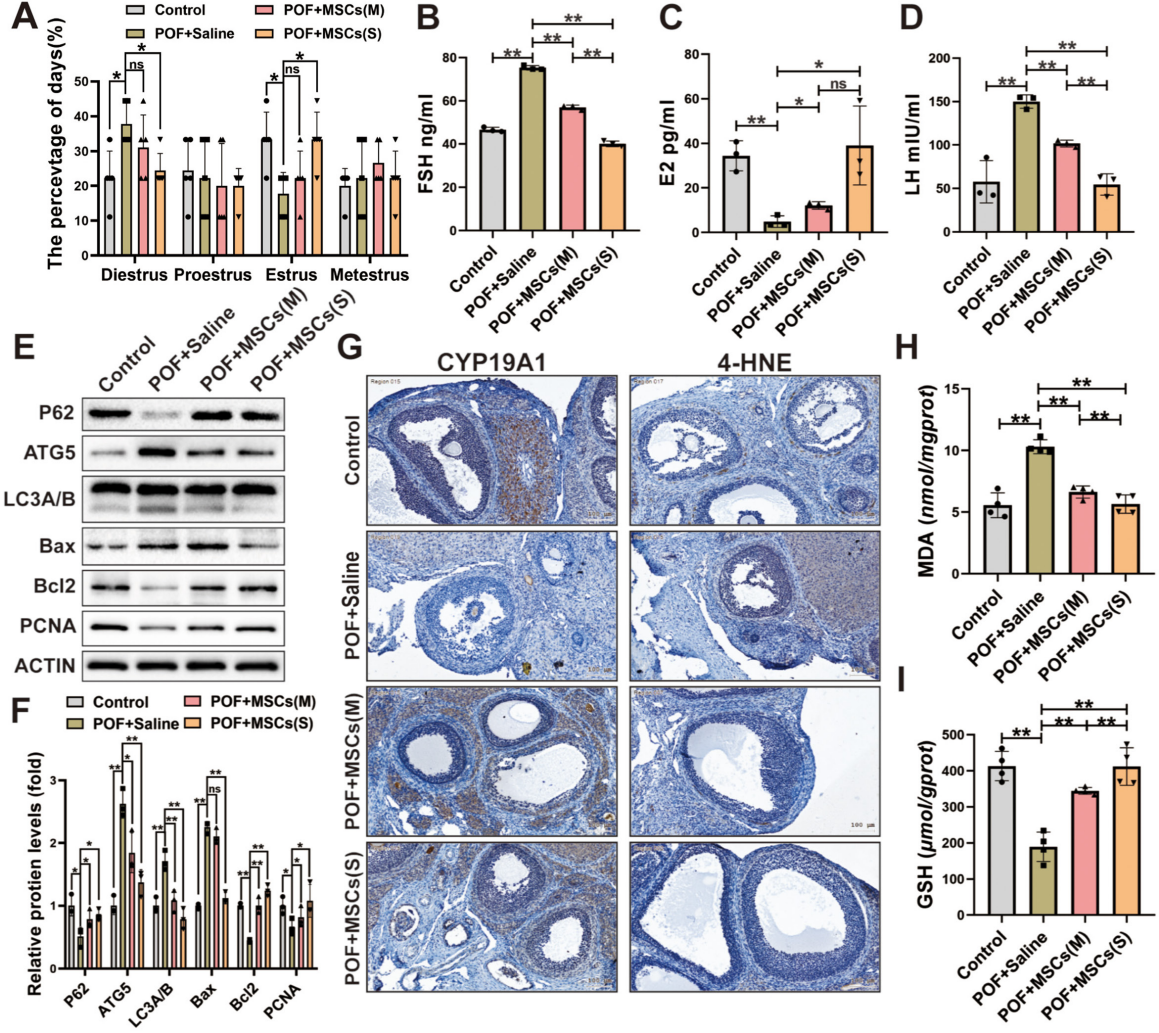
The ovarian function, autophagy, apoptosis, and oxidative stress of ovaries in the POF rat model were improved after hUC-MSCs transplantation. (A) Comparative analysis of estrous cycle markers was conducted among the four groups (*n* = 5). The levels of FSH (B), E2 (C), and LH (D) were measured after hUC-MSCs transplantation (*n* = 3). (E) Western blotting images of autophagy-related proteins (P62, ATG5, LC3A/B), apoptosis-related proteins (Bax, Bcl-2), and proliferation-related protein (PCNA) expression were examined. (F) The quantification histogram (E) was created. (G) Immunohistochemistry images of granulosa cell function-related protein (CYP19A1) and lipid peroxidation marker (4-HNE) in the ovary were analyzed. The oxidative stress markers MDA (H) and GSH (I) in the ovaries were measured (*n* = 4). *P < 0.05, **P < 0.01. Statistical tests for A and F involved two-way ANOVA followed by Dunnett’s test; for B, C, D, H, and I, one-way ANOVA followed by Dunnett’s test.

The estrus cycle was evaluated by staining vaginal smears in rats, and the results showed the rats in the POF + Saline group had a longer diestrus period and a shorter estrus period than rats in the Control group. After hUC-MSCs transplantation, a significant improvement was observed in the POF + MSCs (S) group, and the administration of hUC-MSCs (S) effectively ameliorated the disruption of the estrous cycle induced by CTX, whereas no significant improvement was observed in the POF + MSCs (M) group compared to the POF + Saline group (Fig. 3A). Additionally, the levels of FSH (Fig. 3B), E2 (Fig. 3C), and LH (Fig. 3D) in the POF + MSCs (M), and POF + MSCs (S) groups were significantly improved compared to the POF + Saline group. Therefore, these results suggest that the efficacy of hUC-MSCspheroids orthotopic transplantation was superior to that of monolayer-cultured hUC-MSCs suspension.

### hUC-MSCs ameliorated apoptosis, autophagy, and oxidative stress in ovarian tissues of the POF rat model

**Figure 4:**
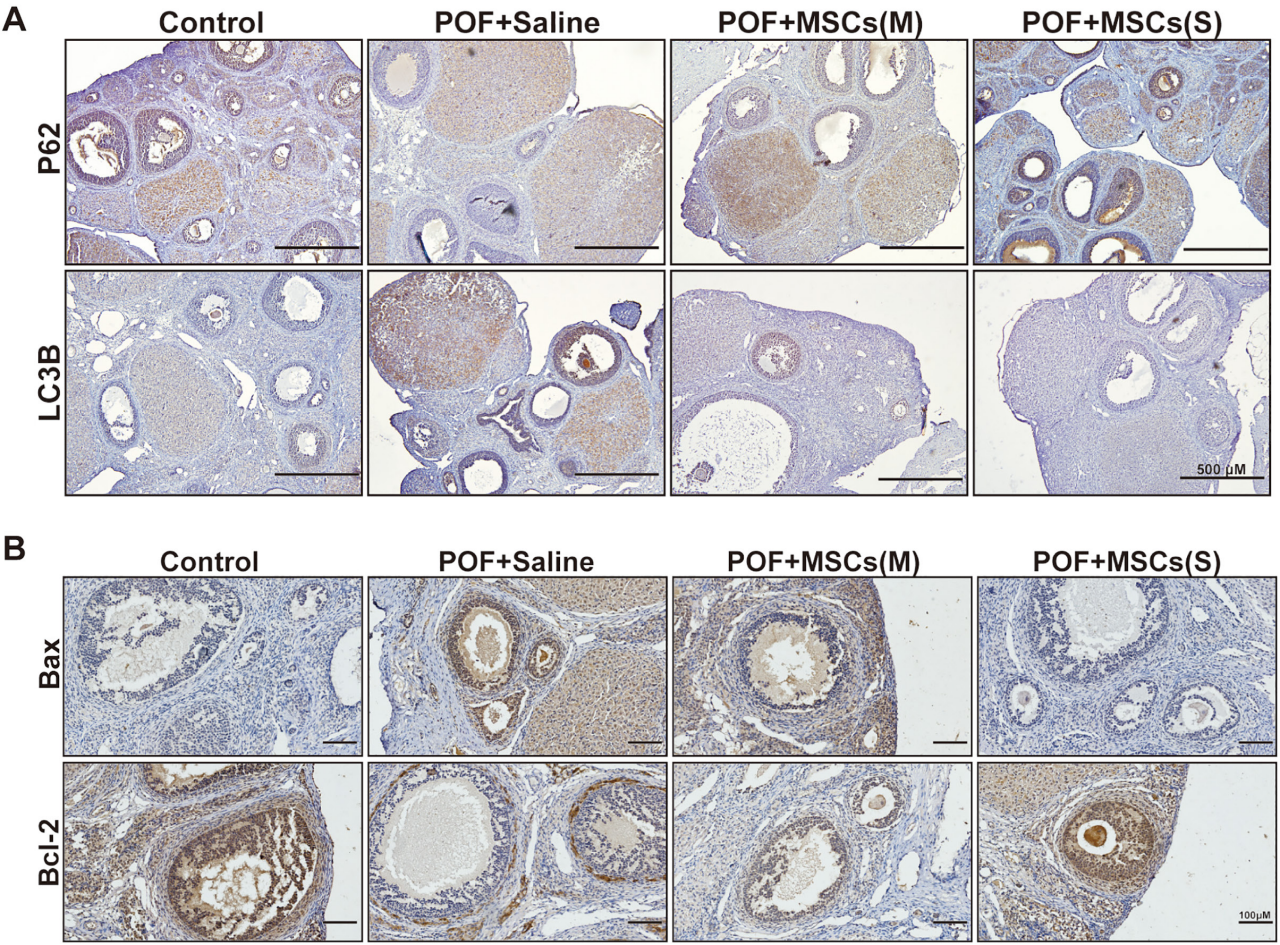
The immunohistochemistry images show the expression of key autophagy-related proteins (P62 and LC3B) and apoptosis-related proteins (Bax and Bcl-2) in ovaries after hUC-MSCs transplantation.

Numerous studies showed that MSCs improved POF models by protecting the ovary from oxidative stress, apoptosis, and autophagy (Yin *et al.* 2018, Lu *et al.* 2019, 2020). In this study, we also compared the effects of two forms of hUC-MSCs on apoptosis, autophagy, and oxidative stress in ovaries after orthotopic transplantation. Then, the expression of key autophagy key proteins (P62, ATG5, and LC3A/B), key apoptosis key proteins (Bcl-2 and Bax), and proliferation-associated protein (PCNA) were detected. The results showed that P62, Bcl-2, and PCNA were increased, while ATG5 and LC3A/B were decreased in the POF+MSCs (M) and POF+MSCs (S) groups compared to the POF+Saline group. However, the expression of Bax was only decreased in the POF+MSCs (S) group (Fig. 3E and F). Additionally, immunohistochemistry results showed that CYP19A1 (functional marker of GCs) was significantly increased, and 4-HNE (lipid peroxidation marker) was decreased in the POF+MSCs (M), and POF+MSCs (S) groups compared to the POF+Saline group (Fig. 3G and Supplementary Fig. 2D). Levels of oxidative stress indicators MDA (Fig. 3H) and GSH (Fig. 3I) in ovarian tissues of each group were compared, and the results showed that they were significantly improved after hUC-MSCs transplantation in the POF+MSCs (M), and POF+MSCs (S) groups, compared to the POF+Saline group. These findings indicated that hUC-MSCs orthotopic transplantation could ameliorate oxidative stress levels and inhibit autophagy and apoptosis in the ovary. Additionally, the expression of key autophagy and apoptosis-related proteins in ovaries was examined by immunohistochemistry. The results showed that P62 and Bcl-2 were increased; LC3B and Bax were decreased in the POF+MSCs (M), and POF+MSCs (S) groups, compared to the POF+Saline group (Fig. 4A, B and Supplementary Fig. 2E). Interestingly, these results revealed that the improvements were mainly focused on the ovary’s granulosa cell layer. Therefore, we hypothesized that hUC-MSCs could reduce the oxidative stress level of the ovary and inhibit autophagy and apoptosis of GCs, thereby restoring ovarian function and improving POF rats.

### hUC-MSC spheroids were superior to hUC-MSCs suspension in inhibiting oxidative damage in KGN cells

**Figure 5:**
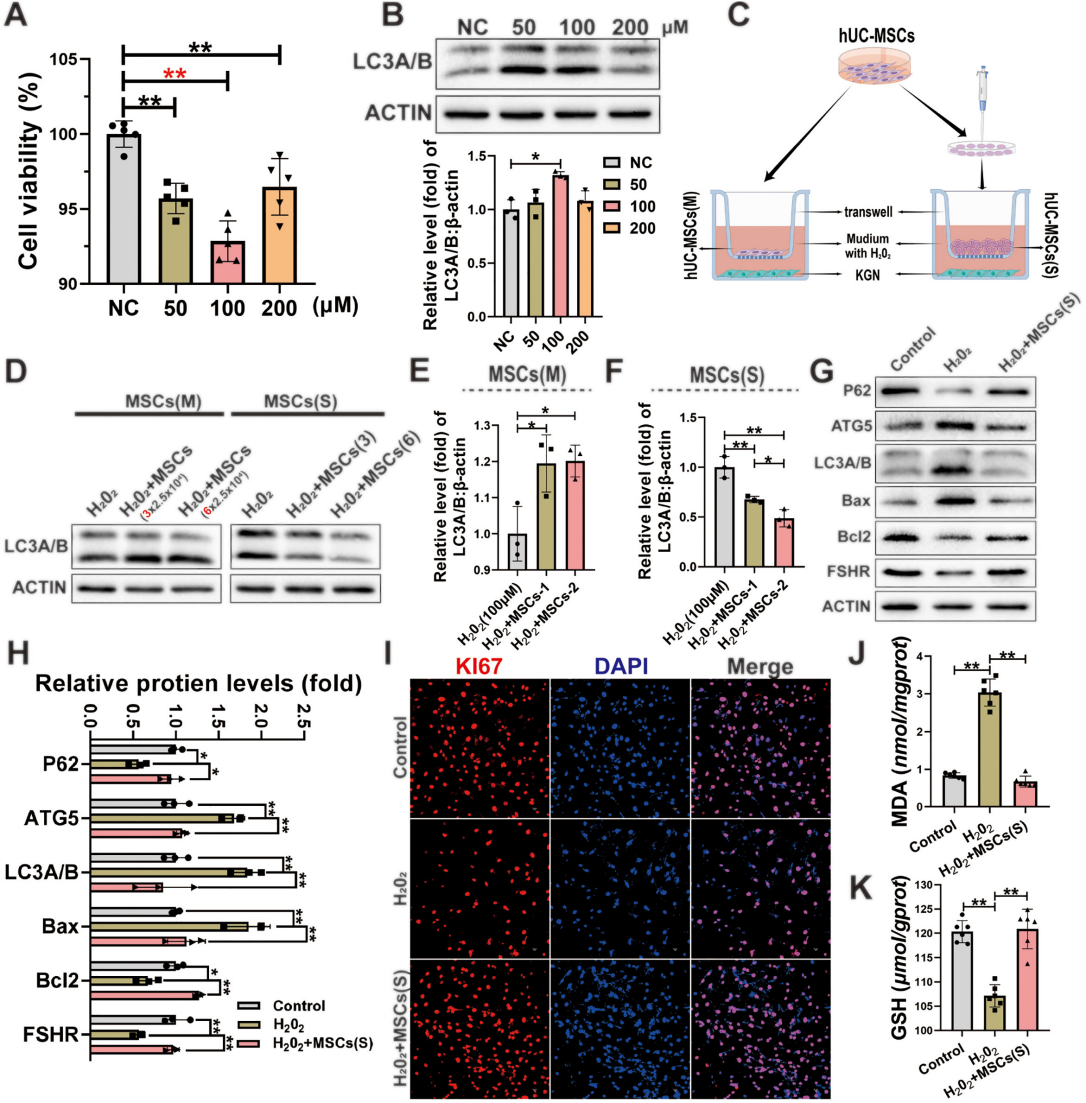
hUC-MSC spheroids exhibited an excellent ability to inhibit H_2_O_2_-induced autophagy and apoptosis in KGN cells. (A) The viability of KGN cells was assessed after exposure to H_2_O_2_. (B) Western blotting images were used to analyze the expression of the autophagy key protein (LC3A/B) after exposure to H_2_O_2_. (C) A schematic diagram of the hUC-MSCs and KGN co-culture system was presented. (D) Western blotting images of LC3A/B in KGN cells were shown after co-culturing with hUC-MSCs (M) and hUC-MSCs (S). (E and F) The quantification histogram for Fig. 6D was provided. (G) Western blotting images displayed the expression of key autophagy-related proteins (P62, ATG5, and LC3A/B), key apoptosis-related proteins (Bax and Bcl-2), and functional protein (FSHR) in H_2_O_2_-induced KGN cells after co-culturing with hUC-MSCs (S). (H) The quantification histogram for Fig. 6G was included. (I) Immunofluorescence images showed the expression of the proliferation-related protein KI67 in H_2_O_2_-induced KGN cells after co-culturing with hUC-MSCs (S). The levels of MDA (J) and GSH (K) were measured in H_2_O_2_-induced KGN cells after co-culturing with hUC-MSCs (S). Statistical tests for H involved two-way ANOVA followed by Dunnett’s test; for A, B, E, F, J, and K, one-way ANOVA followed by Dunnett’s test.

Studies have demonstrated that 3D MSC spheroids have the superior paracrine ability and can better mimic the state of MSCs in the tissue after orthotopic transplantation (Xu *et al.* 2016, Yue *et al.* 2016, Remuzzi *et al.* 2020). In this study, the co-culture system of hUC-MSC spheroids and H_2_O_2_-induced KGN cells was used to investigate the underlying mechanism. First, KGN cells were treated with H_2_O_2_ (50, 100, and 200 µM) for 4 h for optimal exposure conditions. The results showed that the viability of KGN cells was significantly inhibited by all concentrations of H_2_O_2_ (Fig. 5A), and the Western blotting result showed that the expression of autophagy protein LC3A/B was significantly increased by 100 μM H_2_O_2_ (Fig. 5B). Therefore, the optimized condition of 100 μM H_2_O_2_ for 4 h was used for subsequent experiments. To evaluate the recovery of H_2_O_2_-induced KGN cells by hUC-MSCs (M) and hUC-MSCs (S), a transwell co-culture system was established (Fig. 5C). The improvement was evaluated using the protein expression of LC3A/B, and the results showed that hUC-MSCs (S) significantly reduced the expression of LC3A/B; however, the equivalent amount of hUC-MSCs (M) did not significantly reduced it (Fig. 5D, E, F). This result suggested that the effect of hUC-MSC spheroids was superior to the equal number of monolayer-cultured hUC-MSCs in inhibiting H_2_O_2_-induced autophagy in KGN cells.

### hUC-MSC spheroids reduced H_2_O_2_-induced autophagy and apoptosis and improved the function and proliferation of KGN cells

To further confirm the effect of hUC-MSC spheroids on H_2_O_2_-induced GCs or KGN cells, they were co-cultured with hUC-MSC spheroids and analyzed. In the H_2_O_2_+MSCs (S) group, the expression of autophagy-related proteins (P62, ATG5, and LC3A/B), apoptosis-related proteins (Bax and Bcl-2), and the granulosa cell function-related protein (FSHR) significantly improved compared to the H_2_O_2_-induced group (Fig. 5G, H). In addition, to further verify this result, primary GCs of rats were isolated and co-cultured with hUC-MSC spheroids, and then the expression of key autophagy-related proteins (ATG5 and LC3A/B) and apoptosis-related proteins (Bax and Bcl-2) was detected. The results were consistent with those on KGN cells (Supplementary Fig. 2C). Subsequently, the result of immunofluorescence showed that the expression of the proliferation-related protein (KI67) was also significantly restored in the H_2_O_2_+MSCs (S) group compared to the H_2_O_2_-exposed group (Fig. 5I and Supplementary Fig. 2F). Additionally, the result of oxidative stress levels showed that the MDA level in the H_2_O_2_+MSCs (S) group was lower than that in the H_2_O_2_ group (Fig. 5J), and the GSH level was higher than that in the H_2_O_2_ group (Fig. 5K). These results suggested that hUC-MSC spheroids could ameliorate the autophagy and apoptosis of KGN cells caused by oxidative damage.

## Discussion

Numerous studies have demonstrated that hUC-MSCs can significantly improve ovarian function in various POF animal models due to their low immunogenicity (Yang *et al.* 2019, Zhao *et al.* 2020, Deng *et al.* 2021*a*, *b*). The main disadvantage of transvenous transplantation is that the pulmonary vascular system will clear most of the MSCs, resulting in a decrease in the number of MSCs reaching the injured site, making it difficult to achieve a better therapeutic effect. Thus, transvenous transplantation may reduce the planting efficiency of MSCs in the injured ovary. In addition, studies have demonstrated that orthotopic transplantation of hUC-MSCs could effectively improve ovarian function (Ding *et al.* 2018, Yan *et al.* 2020, Igboeli *et al.* 2020). These studies suggest the feasibility of hUC-MSCs to improve POF through orthotopic transplantation in clinical practice.

In this study, we compared the therapeutic effects of monolayer-cultured hUC-MSC suspension and 3D hUC-MSC spheroids by orthotopic transplantation in a POF rat model. The results showed the bodyweight gain was different in the four groups (Control, POF+Saline, POF+MSCs (M), and POF+MSCs (S)). This may be due to the toxicity of CTX in rats, and MSCs can restore the toxic effect (Mo *et al.* 2024). In addition, the results indicated that both cell culture methods had significant effects in restoring ovarian function, but hUC-MSC spheroids exhibited a superior therapeutic effect compared to monolayer hUC-MSCs suspension. Additionally, it has been shown that 3D MSC spheroids are more effective in treating renal ischemia–reperfusion injury (Zhao *et al.* 2016) and ischemic stroke disease models (Li *et al.* 2021*a*,*b*). An increasing number of studies have demonstrated that compared to the monolayer-cultured MSCs suspension, the 3D MSC spheroids have a higher survival rate and greater paracrine capacity, as well as superior stemness maintenance, anti-aging, and anti-inflammation abilities, and promote angiogenesis and tissue repair (Bhang *et al.* 2012, Bartosh *et al.* 2013, Guo *et al.* 2014, Domnina *et al.* 2018). Similar to our findings, Deng *et al.* (2020) demonstrated that MSC spheroids had a better therapeutic effect on spinal cord injury in mice than monolayer-cultured MSCs. Cao *et al.*(2020) confirmed that 3D-cultured MSCs generate more exosomes than monolayer-cultured MSCs. Similar to ours, these results also confirmed that MSC spheroids might have more beneficial effects in treating diseases. Additionally, orthotopic transplantation of MSCs combined with collagen scaffolds restored the ovarian function of mice in GCs, PFs, SFs, and antral follicle atresia (Su *et al.* 2016), which is comparable to our results. Notably, orthotopic transplantation of MSCs significantly reduced autophagy and apoptosis in POF ovarian tissue and alleviated oxidative stress, with GCs reflecting these changes the most. Subsequently, a co-culture system of hUC-MSC spheroids and H_2_O_2_-induced KGN cells was established by transwell* in vitro*, further demonstrated that the antioxidant effects of hUC-MSC spheroids were better than those of hUC-MSCs suspension.

In summary, reasons for the therapeutic advantages of 3D hUC-MSC spheroids may include: (1) the histocompatibility of MSC spheroids after implantation in tissues is enhanced, unaffected by MSCattachment to the substrate (Li *et al.* 2015); (2) A hypoxic environment is formed inside MSCspheroids, which stimulates the secretion of vascular endothelial growth factor (VEGF) and other trophic factors (VEGF, HGF, EGF, etc.) (Miceli *et al.* 2019, Bartosh *et al.* 2010); (3) 3D spheroids can more effectively replicate the state of MSCs entering tissues *in vivo*, maximizing their therapeutic effect (Edmondson *et al.* 2014, Lee *et al.* 2022). Therefore, orthotopic transplantation of hUC-MSC spheroids could be a promising clinical strategy for POF treatment.

Currently, studies have demonstrated that hUC-MSCs primarily improve ovarian function in POF through the following mechanisms: (1) hUC-MSCs secrete angiogenic growth factors like VEGF and promote ovarian vascular remodeling, thereby improving the ovarian microenvironment (Zhang *et al.* 2022). (2) hUC-MSCs reduce oxidative stress in ovarian tissue and provide an excellent environment for follicle development (Deng *et al.* 2021*a*, *b*). (3) hUC-MSCs inhibit autophagy of interstitial membrane cells and restore ovarian function (Lu *et al.* 2020). Reactive oxygen species (ROS) are produced in the mitochondrial respiratory chain process, and moderate ROS facilitate the proliferation and differentiation of GCs, whereas excessive ROS trigger apoptosis and excessive autophagy, leading to follicular development disorders (Dai *et al.* 2023, Zhou *et al.* 2023, Wu *et al.* 2023). A study demonstrated that human placenta-derived MSCs restore ovarian function through an antioxidant effect (Seok *et al.* 2020). Similarly, 3D-cultured placenta-derived MSC spheroids could enhance ovarian function by inducing folliculogenesis (Kim *et al.* 2018). In addition, a study showed the exosomes from menstrual blood-derived MSCs improved ovarian function by inhibiting follicle apoptosis and increasing GC proliferation. Similar to these findings, MSCs can inhibit apoptosis and increase the proliferation of GCs (Zhang *et al.* 2021). Additionally, a study showed that heme oxygenase-1 (HO-1) expressed in MSCs could restore the ovarian function of the POF mouse model by regulating autophagy and apoptosis of GCs (Yin *et al.* 2020). These findings support our conclusion that 3D hUC-MSC spheroids could reduce apoptosis and excessive autophagy of GCs in improving POF.

However, there are still some limitations to this study: (1) although orthotopic transplantation can increase the planting of hUC-MSC spheroids in ovarian tissues, the damage caused by surgery may influence the evaluation of the effect of hUC-MSCs on improving POF. Therefore, optimizing the orthotopic transplantation method remains to be investigated. (2) Although the anti-oxidation, anti-apoptosis, and anti-autophagy effects of 3D hUC-MSC spheroids were better than those of monolayer cultured hUC-MSCs *in vivo* and* in vitro*; however, the underlying mechanisms still require further investigation. Therefore, further studies are needed to elucidate the major components of MSC spheroids that resist oxidative stress in GCs and improve ovarian function.

In conclusion, this study revealed that orthotopic transplantation of 3D hUC-MSC spheroids is more effective than monolayer-cultured hUC-MSCs in improving POF *in vivo* and *in vitro,* and distinctly reducing the level of oxidative stress through the paracrine effect, thereby preventing apoptosis and autophagy of GCs. These findings provide a basis for the clinical application strategy of hUC-MSCs for POF therapy.

## Supplementary Materials

Supplementary Material

## Declaration of interest

The authors declare that there is no conflict of interest that could be perceived as prejudicing the impartiality of the study reported.

## Funding

This work was supported by the National Natural Science Foundation of Chinahttp://dx.doi.org/10.13039/501100001809 (nos. 81960270 and 82204094), and the Key Research and Development Program of Ningxiahttp://dx.doi.org/10.13039/100016692 (2022BEG03084).

## Ethics statement

The study was approved by the Medical Ethics Committee of Ningxia Medical University (NXMU2019-190).

## Data availability

Data will be made available on a request to the corresponding author.

## Author contribution statement

XF and XP contributed reagents, materials, analysis tools, or data; WD and XF: conceived and designed the experiments and wrote the manuscript; WD and HY conceived and designed the experiments and performed the experiments; BX, TH, LL, ZZ, and LD performed the experiments and analyzed and interpreted the data.
