# Supplementary material for: 3D hUC-MSC spheroids exhibit superior resistance to autophagy and apoptosis of granulosa cells in POF rat model
Source: Reproduction. 2024 Jul 13;168(2):e230496. doi: 10.1530/REP-23-0496 (PMC11301424; doi:10.1530/REP-23-0496)
Supplement: Supplementary Material [file supplementary_material.pdf]

Orthotopic transplantation of hUC-MSCs restored POF rat model

To evaluate the effects of hUC-MSCs on the POF rat model, passage five hUC-MSCs were collected for orthotopic transplantation of cultured monolayer suspension and 3D cultured spheroids. The procedure for preparing hUC-MSCs spheroids is shown in Fig. S1A. After anesthesia, the rats in the POF+MSCs (M) group were injected with hUC-MSCs (1×10^6 cells/20 μL) by orthotopic transplantation into the ovaries, the rats in the POF+ MSCs (S) group were injected with an equal number of hUC-MSCs spheroids, whereas the Control and POF+Saline groups were injected with 20 μL of saline. Subsequent assays were detected after 48 days of hUC-MSCs transplantation (Fig. S1B).

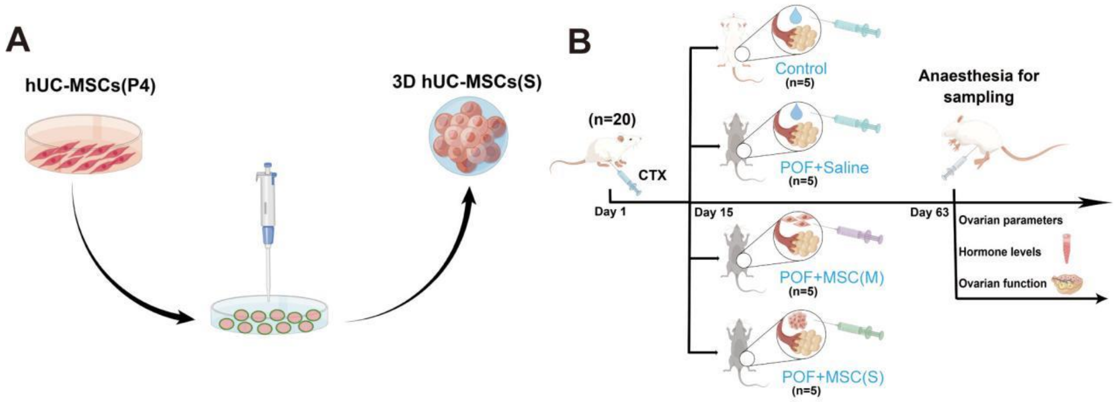

Fig. S1. A: 3D hUC-MSCs spheroids preparation procedure. B: Schematic diagram of animal groups and treatment. (www.figdraw.com)

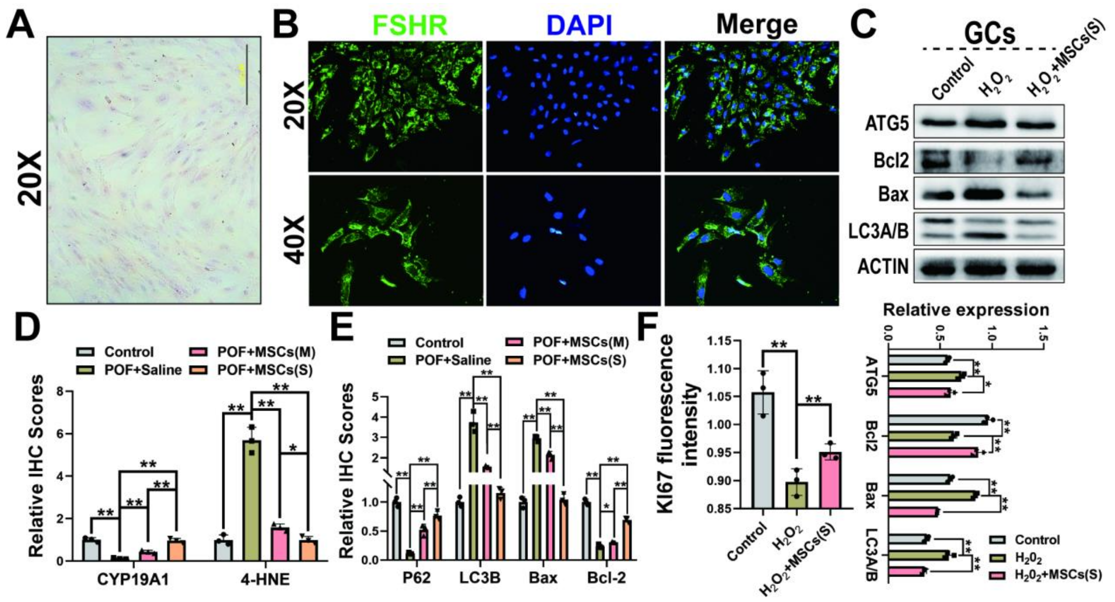

Fig. S2. A: Morphological staining of granulosa cells (GCs). B: Immunofluorescence staining of follicle-stimulating hormone receptor (FSHR) in GCs. C: Western blotting images and quantification histogram showing the expression of autophagy-related proteins (ATG5 and LC3A/B) and apoptosis-related proteins (Bax and Bcl-2) in H_2O_2-induced GCs after co-culturing with hUC-MSCs (S). D: The quantification of CYP19A1 and 4-HNE expression in the ovaries of Figure 4G. E: The quantification of autophagy-related proteins (P62 and LC3B) and apoptosis-related proteins (Bax and Bcl-2) expression in the ovaries of Figure 5. F: Calculation of the rate of KI67-positive cells from Figure 6I. Statistical tests in this figure: C, D, and E were analyzed using two-way ANOVA followed by Dunnett's test; F was analyzed using one-way ANOVA followed by Dunnett's test.

Source of primary antibodies

ATG5 (10181-2-AP) was purchased from Proteintech Co., Ltd., USA; P62 (ab109012) was purchased from Abcam; LC3A/B (#12741) was purchased from Cell Signaling Technology Co., Ltd., USA; Bax (AB026), Bcl-2(AF6285) and β-actin (AF0003) were purchased from Beyond Biotech Co. Ltd., China; PCNA (10205-2-AP) and FSHR (22665-1-AP) were purchased from Proteintech Co., Ltd., China.
